# Association between Knowledge-Attitude-Practices and Control of Blood Glucose, Blood Pressure, and Blood Lipids in Patients with Type 2 Diabetes in Shanghai, China: A Cross-Sectional Study

**DOI:** 10.1155/2017/3901392

**Published:** 2017-12-31

**Authors:** Hua Yang, Jian Gao, Limin Ren, Shuyu Li, Zhangyan Chen, Junfang Huang, Shanzhu Zhu, Zhigang Pan

**Affiliations:** ^1^Department of General Practice, Zhongshan Hospital, Fudan University, Shanghai, China; ^2^Department of Nutrition, Zhongshan Hospital, Fudan University, Shanghai, China; ^3^Center of Clinical Epidemiology and Evidence-Based Medicine, Fudan University, Shanghai, China; ^4^Department of General Practice, Shenzhen Longhua District Central Hospital, Shenzhen, Guangdong, China

## Abstract

Knowledge-attitude-practices (KAP) significantly impact the outcome of self-management in patients with diabetes, yet the association between KAP and the combined control of the levels of blood glucose, blood pressure, and blood lipids in these patients remains uncertain. This community-based cross-sectional study was conducted from December 2014 to December 2016 on 3977 patients with type 2 diabetes in Shanghai. KAP were evaluated using the modified Chinese version of the Diabetes, Hypertension and Hyperlipidemia (DHL) Knowledge Instrument, Diabetes Empowerment Scale–Short Form (DES-SF), and Summary of Diabetes Self-Care Activities (SDSCA). Clinical and biochemical measurements were performed at each sampling site. The association between KAP scores and achieving the combined target goal was assessed by multiple logistic regression. Patients having a higher score of knowledge were more likely to achieve the combined target goal. Furthermore, a turning point of knowledge score was found that the possibility of achieving the combined target goal presented a sharp increase when the knowledge score was more than 70. However, the scores of attitude and practices had no significant relations with achieving the combined target goal. Health intervention strategies, especially increasing integrated diabetes knowledge, should be targeted to patients with type 2 diabetes in communities.

## 1. Introduction

Diabetes mellitus, one of the most common noncommunicable diseases, brings enormous burden not only to individuals but also to national healthcare systems [1]. According to the report of the International Diabetes Federation in 2015 [2], China had the largest number of patients with diabetes mellitus (109.6 million) in the world, and its diabetes-related health expenditure (51 billion USD) ranked only second to the United States.

Several studies have demonstrated that a good control of blood glucose, blood pressure, and blood lipids could prevent or delay the complications of diabetes, reduce diabetes-related health expenditure, and elevate the quality of life of patients with diabetes [3–7]. However, these target goals were not satisfactorily controlled worldwide [8–10]. Most of the studies focused on the relationship between knowledge-attitude-practices (KAP) and the level of fasting blood glucose (FBG) or hemoglobin Alc (HbA1c), and different studies presented contradictory results [11–13]. Meanwhile, hypertension and dyslipidemia are common comorbidities of diabetes. Most previous studies [14–17] demonstrated that controlling blood pressure and blood lipids to target goals could reduce the risk of macrovascular and microvascular complications in patients with diabetes. To date, however, the association between KAP and the control of blood pressure and blood lipids in patients with diabetes has rarely been assessed, and even the association between KAP and achieving the combined target goal has not been reported yet in this population.

This cross-sectional study assessed the diabetes-related knowledge, attitude, and practices of patients with diabetes in the six communities in Shanghai. Furthermore, it explored the association between KAP and achieving the combined target goal for the control of blood glucose, blood pressure, and blood lipids in this population.

## 2. Materials and Methods

The present assessment of the association between KAP and achieving the combined target goal for the control of blood glucose, blood pressure, and blood lipids in patients with type 2 diabetes mellitus (T2DM) was based on a cross-sectional study conducted in six communities in Shanghai.

### 2.1. Population

In this cross-sectional study, conducted between December 2014 and December 2016 in Shanghai, a stratified random sampling procedure was employed to recruit participants. Three districts, including Xuhui from old central ones, Pudong from new central ones, and Jiading from fringing ones, were purposefully selected from 16 districts in Shanghai. In each of these three districts, two community health centers (CHCs) were randomly selected, hence including Kangjian, Xujiahui, Huamu, Weifang, Gongyequ, and Huangdu CHCs; the participants were randomly recruited from these six CHCs. The inclusion criteria for the study population were 18 years of age or older, being diagnosed with T2DM ≥ 3 months previously, being incorporated into the diabetes mellitus management system of China, and no pregnancy. Written informed consent was obtained from all volunteers. The study protocol was approved by the Ethics Committee of the Zhongshan Hospital, Fudan University (B2016-029).

### 2.2. Measurements

From each CHC, the participants were interviewed face to face by the trained investigators, including general practitioners (GPs), public health physicians, and nurses. The survey questionnaire containing demographic information and self-reported scales on diabetes knowledge, attitude, and practices of participants was individually administered under the supervision of investigators. Assistance was provided to those who had difficulty completing the questionnaire primarily due to poor vision. Basic information of participants included age, gender, educational attainment, marital status, smoking status, family history of diabetes mellitus, duration of diabetes mellitus, current medical treatment, and medical history of hypertension, coronary heart disease, and stroke diagnosed by physicians. Clinical and biochemical measurements including body weight, height, blood pressure(measured on the spot three times after resting for more than 10 min and then the average taken), FBG, HbA1c, total cholesterol (TC), triglyceride(TG), low-density lipoprotein cholesterol (LDL-C), high-density lipoprotein cholesterol (HDL-C), blood urea nitrogen (BUN), creatinine (CRE), uric acid (UA), urinary albumin to creatinine ratio (UACR), and urine routine were performed in each sampling site under a fasting condition of participants in the morning. Nonmydriatic fundus photography was implemented using the standard vision digital color fundus cameras (Smartscope PRO from Optomed Oy, Oulu, Finland, or Canon CR-2 from Canon Inc.) by trained GPs. At least two-field retinal photographs based on macula fovea as the center were taken according to a standard protocol.

### 2.3. Assessment of Variables

#### 2.3.1. Knowledge-Attitude-Practice Evaluation

A Chinese modified version of the Diabetes, Hypertension and Hyperlipidemia (DHL) Knowledge Instrument[18] was employed to assess the knowledge of participants, which contained 20 items on 4 dimensions in relation to the knowledge of diabetes, hypertension, hyperlipidemia, and medication and required about 10–15 min to administer. Each item of the questionnaire was scored by the patients as true, false, or do not know, and 1 point was allocated for a correct response and 0 point for an incorrect or “do not know” response. The total score was converted into a hundred percentage point from 0 to 100 [19]. The higher scores indicated a good knowledge of participants.

A Chinese version of Diabetes Empowerment Scale–Short Form (DES-SF) [20] was employed to assess the attitude of participants, which contained eight items and required 5–10 min to administer. Each item of the questionnaire was scored by the patient as strongly agree, agree, neutral, disagree, and strongly disagree, and 1 to 5 points were allocated for each response [21]. The mean score of all items directly reflected the empowerment of participants. The higher scores indicated a good empowerment of participants.

A Chinese version of the Summary of Diabetes Self-Care Activities (SDSCA) measure [22] was employed to assess the practices of participants, which contained 12 items assessing the following aspects of the diabetes regimen over the previous 7 days: general diet, specific diet, exercise, blood glucose testing, foot care, medications, and smoking. Except the smoking item, other items were scored from 0 to 7 points, which presented the achieved self-management in the previous 7 days. The total score was converted into a hundred percentage point from 0 to 100 [23]. The higher scores indicated good practices of participants.

#### 2.3.2. Control of Blood Glucose, Blood Pressure, and Blood Lipids

The levels of HbA1c, systolic and diastolic blood pressure, and TC, TG, LDL-C, and HDL-C presented the target goals for the control of blood glucose, blood pressure, and blood lipids, respectively. According to the Chinese diabetes guidelines (2013 edition) [24], HbA1c < 7%, blood pressure < 140/80 mmHg, and blood lipids meeting the standard of TC < 4.5 mmol/L, TG < 1.7 mmol/L, LDL-C < 2.6 mmol/L (without coronary heart disease), LDL-C < 1.8 mmol/L (with coronary heart disease), HDL-C > 1.0 mmol/L(male), and HDL-C > 1.3 mmol/L (female) indicated achieving blood glucose, blood pressure, and blood lipid target goals, respectively.

### 2.4. Statistical Analysis

Statistical analysis was carried out using SPSS software, version 17.0 (SPSS Inc., IL, USA) and SAS software, version 9.2 (SAS Institute, NC, USA).

The data were presented as percentages or means and standard deviations. Significant differences between groups were calculated using chi-square tests, corrected chi-square tests, or Fisher's exact test for the percentages and unpaired *t*-tests for the mean values. The association between KAP and achieving the combined target goal for the control of blood glucose, blood pressure, and blood lipids was assessed via multiple logistic regression. The multiple logistic regression models were fitted using achieving or not achieving the combined target goal as the dependent variables and the quartiles of KAP scores as the independent variables. The first quartile of KAP scores was used as reference in this model. Adjustments were made for confounding factors, including age, gender, educational attainment, marital status, smoking status, family history of diabetes mellitus, duration of diabetes mellitus, and current medical treatment. The odds ratios (ORs) and 95% confidence intervals (CI) were calculated. A two-tailed alpha with *P* < 0.05 was considered statistically significant for all analyses.

## 3. Results

### 3.1. Characteristics of Participants

**Figure 1:**
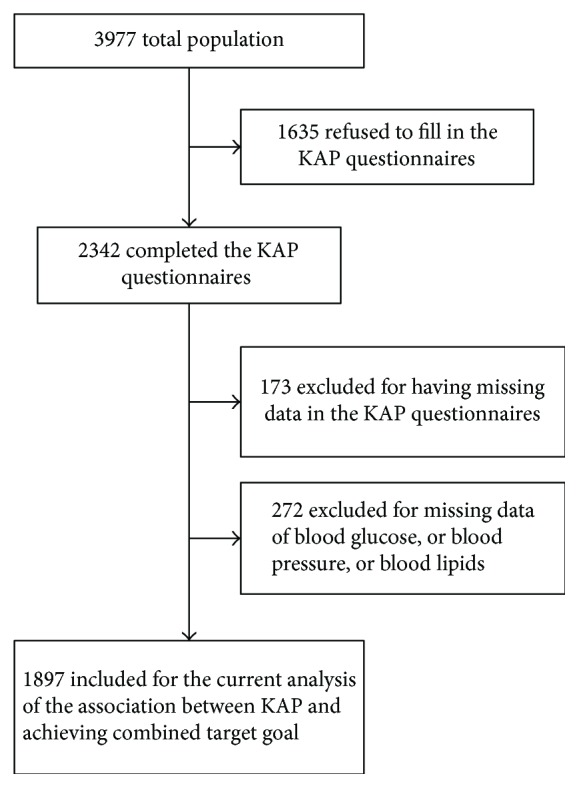
Inclusion/exclusion criteria for the current study participants for the assessment of the association between KAP and achieving combined target goal.

In a total of 3977 participants without missing basic sociodemographic characteristics data, 47.7% who completed the KAP questionnaire without missing data and underwent the measurement of blood glucose, blood pressure, and blood lipids were included in the present study (Figure 1).

It was found that 42.6% of participants were male; 19.7% were current smoker; average duration of diabetes mellitus was 9.2 years; 76.5% were treated with oral hypoglycemic medicines, 8.3% with insulin, 7.5% with oral hypoglycemic medicine and insulin, and 7.6% without medicine; 75.0% had comorbid hypertension, 8.9% comorbid coronary heart disease (CAD), and 6.9% comorbid stroke; 25.0% had microalbuminuria; 3.5% had macroalbuminuria; and 18.8% had diabetic retinopathy (DR). Although 48.7%, 34.1%, and 15.6% achieved the target goals for the control of blood glucose, blood pressure, and blood lipids, respectively, only 2.9% achieved the combined target goal (Table 1).

On average, the male participants were more likely to achieve the combined target goal compared with the female ones (*P* < 0.05). Significant differences were observed between sociodemographic and clinical characteristics of those who achieved the combined target goal and those who did not achieve the combined target goal. A higher proportion of participants with the higher level of education, diabetes family history, without hypertension, BMI < 24 kg/m^2^, or negative UACR achieved the combined target goal (*P* < 0.05) (Table 1).

### 3.2. KAP Scores of Participants

Of the 1897 participants' KAP scores in the present study, the total score of knowledge was 65.9 ± 20.0, and knowledge of diabetes mellitus got the highest score of 76.3 ± 23.9, whereas knowledge of blood lipids got the lowest score of 39.5 ± 31.1; the average score of attitude was 3.7 ± 0.8; the total score of practices was 47.6 ± 14.3, and the practice of general diet got the highest score of 82.3 ± 27.3, whereas the practice of blood glucose testing got the lowest score of 14.0 ± 20.8.

The Spearman correlation analysis indicated that the correlation coefficients between the score of knowledge and attitude, knowledge and practices, and attitude and practices were 0.203, 0.107, and 0.150, respectively (*P* < 0.001).

Compared with the patients with T2DM who did not achieve the combined target goal, those who achieved the combined target goal had significantly increased total and subscale scores of knowledge and a total score of practices (*P* < 0.05; Table 2).

### 3.3. Association between KAP Scores and Achieving Combined Target Goal

The results from the multiple logistic regression analysis are presented in Table 3.

Patients with T2DM having a higher score of knowledge were more likely to achieve the combined target goal. On adjusting age, gender, educational attainment, marital status, family history of diabetes mellitus, duration of diabetes mellitus, current medical treatment, and smoking status, those whose knowledge score was in the highest quartile had 5.5-fold increases (OR = 6.519, 95% CI 2.196–19.347, *P* for trend < 0.001) in the possibility of achieving the combined target goal compared with those in the lowest quartile. However, attitude and practice scores had no significant relations with achieving combined target goals.

**Figure 2:**
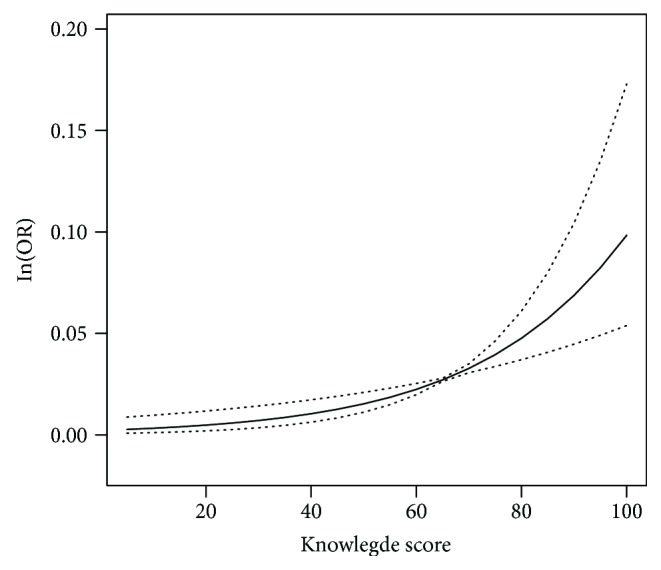
Curve fitting between knowledge score and ln(OR). OR for achieving combined target goal associated with an increase in knowledge score. Adjusted for age, gender, educational attainment, marital status, family history of diabetes mellitus, duration of diabetes mellitus, current medical treatment, and smoking status. The dotted line presented 95%CI.

Furthermore, a threshold effect analysis was conducted. A turning point of 70 in the *x*-axis was observed. The curve presented a sharp increase when the knowledge score was more than 70 (*P* = 0.001), and a temperate increase was presented when the knowledge score was less than 70 (*P* = 0.185) (Figure 2).

## 4. Discussion

This cross-sectional study presented the positive association between having more knowledge about diabetes and attaining the combined target goal for the control of blood glucose, blood pressure, and blood lipids in patients with T2DM in communities of Shanghai. In the present sample, the knowledge on diabetes, hypertension, and medication was well mastered by patients with T2DM in communities in Shanghai, with all scores higher than 70 out of 100. However, the knowledge of hyperlipidemia was deficient, with the score less than 40. Previous reports of evaluating the knowledge of patients with diabetes usually focused on diabetes knowledge. de Oliveira et al. [25] investigated 79 patients with diabetes in a primary health unit in Brazil using The Diabetes Mellitus Knowledge questionnaire and found that only 35.4% of patients presented good knowledge on diabetes. Zhang et al. [26] reported that 38.7% of patients with T2DM in communities had low-level knowledge on diabetes. In Lai et al.'s study [19], which used the DHL knowledge instrument to assess the knowledge of patients on diabetes, hypertension, hyperlipidemia, medication, and general issues, results similar to those of the present study were obtained, showing that the score of hyperlipidemia knowledge was the lowest. Generally speaking, the residents of Shanghai, a developed area in terms of economy and culture in China, have more access to various knowledge and information. However, the low level of knowledge on hyperlipidemia can be explained as that the management of diabetes mellitus and hypertension was included in the National Basic Public Health Service Management Project since 2009, and patients with T2DM were regularly followed up by primary medical workers in CHCs [27] and obtained organized diabetic education frequently. However, hyperlipidemia was not included in the National Basic Public Health Service Management Project yet and gained less attention of patients and primary medical workers.

The patients with T2DM got the highest score in the general diet of self-care activities, but the lowest score in blood glucose testing. Similar to the present study, Freitas et al. [28] observed that 92.7% of patients with diabetes did not monitor blood glucose correctly. In the study of diabetic outpatients, Kueh et al. [29] found that frequent self-monitoring of blood glucose would increase the anxiety and depression in patients, which reflected the rejection of blood glucose monitoring by patients from another perspective.

In the present study, 48.7% of patients achieved the target goal for the control of blood glucose, which was similar to the findings of Ji et al.'s study [10]. However, a higher rate of achieving the target goal for the control of blood pressure and a lower rate of achieving the target goal for the control of blood lipids were observed in the present study compared with those in Ji et al.'s study. The discrepancy might be because the target blood pressure level recommended in the new guideline of China was used [24] and the target goal for the control of blood lipids was set as achieving TC, TG, LDL-C, and HDL-C goals all together. Therefore, only 2.9% of patients achieved the combined target goal in the present study. Previous studies reported [30–32] that not only TC but also TG, LDL-C, and HDL-C were the risk factors for arteriosclerotic cardiovascular disease. Hence, a more comprehensive assessment of blood lipids in patients with diabetes would be helpful for preventing or delaying diabetes-related complications.

In terms of the association between KAP and the levels of blood glucose, blood pressure, and blood lipids in patients with diabetes, different studies showed different results. Ghannadi et al. [33] found a significantly negative correlation between knowledge and attitude with HbA1c level and FBG in patients with diabetes on dialysis. Wang et al. [34] investigated patients with T2DM in Yakeshi, a remote city in northern China, and found that nutritional education was effective in improving patients' nutrition knowledge and practices, and this optimal practice helped patients control blood glucose effectively. Yang et al.'s study [35] indicated that perceived diabetes empowerment was a predictor of self-care behavior and HbA1c in Chinese patients with T2DM. However, Fitzgerald et al. [36] failed to find any statistically significant relationship between the empowerment of patients with diabetes and blood glucose or blood lipid control in Ireland communities. Nevertheless, no study was conducted on KAP related to achieving the combined target goal, including blood glucose, blood pressure, and blood lipids, at the community level. In the present study, a positive association was found between the patient's knowledge level and achieving the combined target goal. Furthermore, an interesting finding was that the benefit of the knowledge score was outstanding till the score increased to a specific value, indicating that the primary care workers should increase patient's knowledge to a score more than 70 when using the Chinese modified version of the DHL Knowledge Instrument in the health education program. In addition, the results highlighted that healthcare providers should pay more attention to elevating the knowledge level via various ways, which may benefit the health outcome in patients with T2DM. Not only diabetes knowledge but also hypertension and hyperlipidemia knowledge was equally important in attaining the integrated goal in diabetes management. The results of the present study indicated little evidence showing a direct linear relationship of patient's attitude and practices, as measured by DES-SF and SDSCA, respectively, with achieving the combined target goal. Theoretically speaking, patients' attitude, especially practices, vitally accounted for the health outcome. However, the necessary and sufficient conditions for the change to occur were unknown in the real world [36].

A limitation of this study concerned the representativeness of the study sample. In the present study, most of the participants were elderly people, who played a predominant role in the real world of diabetes management in CHCs. Self-reported physician-diagnosed chronic diseases might have resulted in some misclassification and information bias. Moreover, this study was cross-sectional in design. Therefore, only association, rather than causation, could be evaluated. Longitudinal studies will be required to better elucidate the cause–effect relationships between KAP and health outcome in this population. Further research into the short- and long-term health outcomes of KAP evaluation will be needed to explore their value in diabetes management.

## 5. Conclusions

Patients with T2DM who had more knowledge about diabetes were more likely to attain the combined target goal for the control of blood glucose, blood pressure, and blood lipids. In light of this result, health intervention strategies, especially elevating integrated diabetes knowledge, should be targeted to patients with T2DM in communities.

## Figures and Tables

**Table 1:** Prevalence of achieving the combined target goal in patients with T2DM by sociodemographic and clinical characteristics.

Variables	Total (*N*^§^ = 1897)	Achieving the combined target goal (*N*^§^ = 55)	Not achieving the combined target goal (*N*^§^ = 1842)	*χ* ^2^ or *t*	*P* value^△^
Age (year), mean ± SD	67.4 ± 8.4	68.9 ± 7.4	67.3 ± 8.4	1.330	0.184^∗^
Gender, *N* (%)					
Male	808	36 (4.5)	772 (95.5)	12.107	0.001^#^
Female	1089	19 (1.7)	1070 (98.3)		
Educational attainment, *N* (%)					
Elementary or lower	692	8 (1.2)	684 (98.8)	14.671	0.002^#^
Junior high school	781	26 (3.3)	755 (96.7)		
High school	262	12 (4.6)	250 (95.4)		
College	162	9 (5.6)	153 (94.4)		
Marital status, *N* (%)					
Single, divorced, or windowed	106	3 (2.8)	103 (97.2)	0.000	1.000^#^
Married or cohabitating	1791	52 (2.9)	1739 (97.1)		
Smoking status, *N* (%)					
Previous smoker/never smoked	1524	44 (2.9)	1480 (97.1)	0.004	0.949^#^
Current smoker	373	11 (2.9)	362 (97.1)		
Family history of diabetes mellitus, *N* (%)					
No	1274	29 (2.3)	1245 (97.7)	6.967	0.008^#^
Yes	522	24 (4.6)	498 (95.4)		
Duration of diabetes mellitus (year), mean ± SD	9.2 ± 6.0	9.3 ± 6.5	9.2 ± 6.0	0.157	0.876^∗^
Onset age (year), mean ± SD	58.3 ± 9.1	59.7 ± 8.5	58.2 ± 9.1	1.129	0.259
Current medical treatment, *N* (%)					
Without medicine	137	3 (2.2)	134 (97.8)		0.731^#^
Oral medicine	1375	38 (2.8)	1337 (97.2)	1.293	
Insulin	150	6 (4.0)	144 (96.0)		
Oral medicine + insulin	135	5 (3.7)	130 (96.3)		
Hypertension, *N* (%)					
No	474	21 (4.4)	453 (95.6)	5.262	0.022^#^
Yes	1423	34 (2.4)	1389 (97.6)		
CAD, *N* (%)					
No	1728	52 (3.0)	1676 (97.0)	0.452	0.501^#^
Yes	169	3 (1.8)	166 (98.2)		
Stroke, *N* (%)					
No	1767	50 (2.8)	1717 (97.2)	0.505	0.444^#^
Yes	130	5 (3.8)	125 (96.2)		
BMI (kg/m^2^), *N* (%)					
<18.5	18	1 (5.6)	17 (94.4)		0.001^#^
≥18.5, <24	599	30 (5.0)	569 (95.0)	15.187	
≥24	1260	23 (1.8)	1237 (98.2)		
SP, mean ± SD	135.5 ± 15.5	122.2 ± 10.3	135.9 ± 15.5	−9.544	<0.001^∗^
DP, mean ± SD	79.6 ± 8.4	72.5 ± 4.6	79.8 ± 8.4	−11.238	<0.001^∗^
HbA1c (%), mean ± SD	7.3 ± 1.5	6.2 ± 0.4	7.3 ± 1.5	−16.932	<0.001^∗^
TC (mmol/L), mean ± SD	4.8 ± 1.0	3.8 ± 0.5	4.9 ± 1.0	−14.953	<0.001^∗^
TG (mmol/L), mean ± SD	1.6 ± 1.2	0.9 ± 0.3	1.7 ± 1.2	−15.222	<0.001^∗^
LDL-C (mmol/L), mean ± SD	2.8 ± 0.9	1.9 ± 0.5	2.8 ± 0.9	−12.773	<0.001^∗^
HDL-C (mmol/L), mean ± SD	1.4 ± 0.4	1.5 ± 0.3	1.4 ± 0.4	2.830	0.005^∗^
BUN (mmol/L), mean ± SD	5.6 ± 1.7	5.5 ± 1.3	5.6 ± 1.7	−0.650	0.516^∗^
SCr (umol/L), mean ± SD	68.6 ± 24.6	70.3 ± 19.9	68.5 ± 24.7	0.516	0.606^∗^
UA (umol/L), mean ± SD	307.9 ± 76.3	298.7 ± 69.2	308.1 ± 76.5	−0.881	0.379^∗^
eGFR^a^ (mL·min^−1^ · (1.73 m^2^)^−1^), mean ± SD	97.7 ± 28.5	101.1 ± 30.3	97.6 ± 28.5	0.872	0.383^∗^
UACR^b^ (mg/g), *N* (%)					
<30	1194	43 (3.6)	1151 (94.4)		0.011^#^
30–300	418	4 (1.0)	414 (99.0)	8.646	
>300	58	1 (1.7)	57 (98.3)		
DR^c^, *N* (%)					
No	515	12 (2.3)	503 (97.7)	0.455	0.500^#^
Yes	119	1 (0.8)	118 (99.2)		

BMI: body mass index; CAD: coronary heart disease; DR: diabetic retinopathy; eGFR: estimated glomerular filtration rate; UACR: urinary albumin creatinine ratio. ^a^eGFR was calculated from the Modification of Diet in Renal Disease formula, as follows: eGFR (mL·min^−1^ · (1.73 m^2^)^−1^) = 186 × CRE (mg/dL)^−1.154^ × age^−0.203^ (× 0.742, if female). ^b^UACR was measured on a single random urine sample and calculated from urinary albumin–creatinine ratio. ^c^DR was defined on the international Clinical Grading Standards of Diabetic Retinopathy (2002) by the ophthalmologist according to the retinal photographs. The results were categorized into two levels: with DR or without DR. ^§^The number of respondents included in the study. ^△^Comparing participants under the combined control of blood glucose, blood pressure, and blood lipids with those out of combined control. ^∗^Using the *t*-test. ^**#**^Using chi-square test.

**Table 2:** KAP scores of patients with T2DM who achieved or did not achieve the combined target goals.

Variables		Total (*N* = 1897)	Achieving the combined target goal (*N* = 55)	Not achieving the combined target goal (*N* = 1842)	*t*	*P* value^△^
Knowledge	Diabetes	76.3 ± 23.9	88.4 ± 17.9	75.9 ± 23.9	5.022	<0.001
	Hypertension	72.5 ± 30.6	80.6 ± 27.7	72.2 ± 30.7	1.999	0.046
	Hyperlipidemia	39.5 ± 31.1	55.3 ± 30.8	39.0 ± 31.0	3.831	<0.001
	Medication	74.7 ± 26.1	86.5 ± 17.3	74.3 ± 26.2	5.058	<0.001
	Total score	65.9 ± 20.0	78.3 ± 15.3	65.6 ± 20.0	5.986	<0.001
Attitude		3.7 ± 0.8	3.8 ± 0.7	3.7 ± 0.8	0.722	0.471
Practices	General diet	82.3 ± 27.3	84.4 ± 30.2	82.3 ± 27.2	0.573	0.567
	Specific diet	48.8 ± 24.4	47.9 ± 27.6	48.8 ± 24.3	−0.262	0.793
	Exercise	40.1 ± 25.1	45.5 ± 22.7	40.0 ± 25.1	1.766	0.083
	Blood glucose testing	14.0 ± 20.8	15.5 ± 24.9	14.0 ± 20.7	0.524	0.600
	Foot care	36.1 ± 40.4	46.0 ± 43.8	35.8 ± 40.3	1.831	0.067
	Medications	81.3 ± 36.8	87.8 ± 29.7	81.1 ± 37.0	1.639	0.106
	Total score	47.6 ± 14.3	51.5 ± 14.6	47.5 ± 14.3	2.019	0.044

^△^Comparing participants under the combined control of blood glucose, blood pressure, and blood lipids with those out of combined control.

**Table 3:** Association between KAP scores and achieving the combined target goal.

Independent variables	Unadjusted OR (95%CI)	Model 1 OR (95%CI)	Model 2 OR (95%CI)
Knowledge score			
Quartile 1	1	1	1
Quartile 2	1.126 (0.341–3.717)	1.151 (0.348–3.807)	1.336 (0.330–5.405)
Quartile 3	2.964 (1.159–7.577)	2.849 (1.112–7.297)	3.803 (1.251–11.562)
Quartile 4	5.448 (2.229–13.316)	5.238 (2.139–12.838)	6.519 (2.196–19.347)
*P* for trend	<0.001	<0.001	<0.001
Attitude score			
Quartile 1	1	1	1
Quartile 2	1.512 (0.685–3.336)	1.547 (0.699–3.421)	1.681 (1.733–3.851)
Quartile 3	1.445 (0.635–3.287)	1.474 (0.646–3.364)	1.299 (0.535–3.154)
Quartile 4	1.413 (0.621–3.213)	1.371 (0.601–3.128)	1.257(0.529–2.987)
*P* for trend	0.492	0.545	0.859
Practice score			
Quartile 1	1	1	1
Quartile 2	1.736 (0.772–3.905)	1.675 (0.743–3.778)	1.642 (0.697–3.866)
Quartile 3	1.067 (0.449–2.535)	1.021 (0.429–2.431)	1.008 (0.409–2.485)
Quartile 4	2.123 (0.976–4.615)	2.030 (0.931–4.427)	1.778 (0.775–4.080)
*P* for trend	0.137	0.171	0.332

Dependent variable: achieving or not achieving the combined target goal. Model 1: adjusted for age and gender. Model 2: adjusted for age, gender, educational attainment, marital status, family history of diabetes mellitus, duration of diabetes mellitus, current medical treatment, and smoking status.
